# Completion of four or more ANC visits among women of reproductive age in Iganga district in Uganda: a quantitative study on the role of service-level factors

**DOI:** 10.1186/s12913-023-09913-7

**Published:** 2023-08-24

**Authors:** Lorna Barungi Muhirwe, Magdeline Aagard

**Affiliations:** 1 School of Health Sciences, Walden University & Independent Researcher, Kampala, Uganda; 2 Core Faculty, School of Health Sciences, Health Services and DHA, Walden University, Minneapolis, MN USA

**Keywords:** Antenatal care attendance, Service level factors, Service provision

## Abstract

**Background:**

Evidence indicates that antenatal care (ANC) has both indirect and direct effects on maternal and perinatal morbidity and mortality reduction. In Uganda, the ANC attendance rate stands at 97.3% for one visit, but 59.9% for four or more visits. Given the imminent shift to the eight-contact ANC model in Uganda, combined with a lack of universal coverage for completion of four ANC visits, there is need for research that provides information on the factors that differentiate completers of recommended ANC attendances from non-completers. The aim of this quantitative study was to assess service- level factors affecting completion of ANC attendance defined by completion of four or more visits among women of reproductive age in Iganga district in Uganda.

**Methods:**

Facility assessment scores on the service-level factors of interest for health facilities were obtained using a service level index tool. The relationship between the ANC completion rates of clients sampled from records at the health facilities and facility scores on service-level factors of interest were analyzed. Regression was conducted to determine the predictive relationship between ANC service availability, ANC service content, and ANC service organization, and completion of ANC attendance.

**Results:**

The model was statistically significant, χ2 (6) = 26.118, *p* ˂ 0.05, and accounted for approximately 17.3% of the variance of ANC attendance completion (R2 = .173). Completion of ANC attendance was primarily predicted by better timing of provision of ANC services, and to a lesser extent by higher levels of availability of medicines and medical supplies.

**Conclusions:**

This study demonstrated that service-level factors have a predictive value for completion of ANC attendance. The findings can be used to improve availability, content, and organization of ANC services with the aim of enhancing positive experiences for clients and motivating them to complete the recommended number of ANC visits.

**Supplementary Information:**

The online version contains supplementary material available at 10.1186/s12913-023-09913-7.

## Background

Of the 1.5 million babies born in Uganda annually, 38,000 are stillborn [1]. More than half the stillbirths in sub-Saharan Africa are antepartum (occurring during pregnancy before the onset of labor) with the major underlying causes being hypertensive disorders of pregnancy, infections, and placental complications resulting in antepartum hemorrhage [2, 3]. Together, these three conditions account for three quarters of antepartum stillbirths in sub-Saharan Africa for which the cause is known [2]. In Uganda, specifically, hypertensive disorders, anemia, and syphilis are some of the leading maternal conditions associated with stillbirths [4].

Evidence indicates that antenatal care (ANC) has both indirect and direct effects on maternal and perinatal morbidity and mortality reduction [5]. For example, Lawn et al. [6], demonstrated that higher national coverage of ANC is strongly associated with lower antepartum stillbirth rates. Furthermore, 50% of stillbirths and 60% of early neonatal deaths are not linked to specific maternal conditions, underscoring the need for early identification of at-risk pregnancies during the prenatal period [1]. ANC from a skilled provider is a proven intervention aimed at monitoring pregnancy to reduce morbidity and mortality risks for the mother and child that may occur during pregnancy, delivery, and the postnatal period [7]. This is attributed to the opportunity that the ANC platform provides to detect and treat pregnancy-related complications and to ensure early identification and mitigation of risk factors for complications during labor and delivery [5, 8]. In Uganda, the ANC attendance rate stands at 97.3% for one visit but drops to 59.9% for four or more visits [9]. Rural regions in Uganda have rates as low as 58% for four or more visits, indicating noncompletion of up to 42% among pregnant women in rural areas, with the worst performing region in the country having a rate of only 44.5% [9].

Maternal and perinatal survival are currently a high priority for the government of Uganda (GOU). as indicated in the government’s Sharpened Plan and Investment Case for Reproductive, Maternal, Newborn, Child, and Adolescent Health [4]. ANC service provision is part of Cluster 2 of the maternal and child health cluster in the Uganda Minimum Health Care Package for pregnant women in Uganda [10]. While the country is currently implementing a minimum of (at least) four ANC visits, the government has incorporated an eight-contact model into the national sexual and reproductive health policy guideline and in 2018 started initiating activities to support a gradual switch to the eight-contact ANC model recently recommended by WHO for low- and middle-income countries [11]. The eight-contact model emphasizes completion of the first visit as early as possible in the first trimester of pregnancy, with the next visit scheduled at 20 weeks and then repeat visits at 26 weeks, 30 weeks, 34 weeks and then every two weeks until delivery [7]. Given the imminent shift to the eight-contact ANC model of service delivery level in Uganda, combined with a prevailing lack of universal coverage for completion of four ANC visits, there is need for research that provides current information on the factors that differentiate completers of recommended ANC attendances from non-completers, particularly in the rural areas of the country [12].

The most recent national Service Provision Assessment (SPA) survey for Uganda was conducted in 2007 and collected information on overall availability of facility-based services, including ANC services. The 2007 survey data indicated that 70% of health facilities surveyed offered ANC services, but only 20% of health facilities had all the equipment and medical supplies necessary for provision of basic ANC services [13].

Findings from a 2013 survey indicated that 70% of the health facilities visited offered ANC services, but actual provision of discrete components of the recommended ANC service package varied from 69% providing intermittent preventive treatment for malaria in pregnancy to only 12% of health facilities providing advanced distribution of misoprostol to pregnant women [14].

A study conducted in 2018, found that the trend in inconsistent delivery of the comprehensive ANC service content has persisted over a twenty-year period with the lowest overall coverage being having a urine sample taken at 27.9% and the highest overall coverage being having been weighed at 83.6% [12]. Benova et al. [12], also found that only 9.6% of women who completed four or more ANC visits received all eight ANC care components.

Chowfla et al. [15], found that screening, diagnosis, and management of common causes of maternal and neonatal morbidity and mortality during pregnancy was poorly conducted in health facilities surveyed with only three percent of expected cases of pre- eclampsia being diagnosed on a monthly basis.

From the results of the 2007 SPA, 58% of the health facilities provided ANC services for five or more days per week while 37% offered the services for only two days per week. Lower frequency of ANC service provision per week was more likely for primary care facilities than for higher-level and referral facilities such as hospitals [13]. Conrad et al. [16], found gaps in ANC provision in Uganda including ineffective organization of educational sessions, selective omission of certain services, lack of explanation of important clinical and laboratory procedures, failure to link the performed procedures with preventive information, and occasional lack of respect for clients.

Findings from the 2016 DHS in Uganda revealed that 90% of women who gave birth in the five years preceding the survey received ANC from a skilled provider at least once for their last birth [9]. These findings are similar to the National Service Delivery survey data that indicated 97% of women who needed ANC services utilized them [17]. However, nationally only 60% of women received ANC from a skilled provider at least four times [9].

Kawungezi et al. [18] and Bbaale [19], focused mainly on the role of non-facility level factors on ANC utilization. Kawungezi et al. [18], found that individual level factors such as religion, occupation, level of education, and parity influenced place of ANC attendance, number of ANC visits, and ANC booking time in Uganda. Bbaale [19], also found significant associations between individual level factors such as regional disparities, religion, access to media, maternal decision-making autonomy, wealth status, timing of pregnancy, birth histories, birth order, and frequency, and timing of ANC attendance in Uganda [18]. Bbaale [19], strengthened the evidence that women in rural areas of Uganda have lower utilization rates of ANC compared to their urban counterparts.

Studies on ANC service utilization in Uganda have focused on ANC service availability, service content, and to a limited extent service organization. Few studies have focused on the relationship if any, between these factors and ANC service utilization defined by completion of the recommended ANC visits.

## Methods

### Study design and setting

This was a quantitative study involving investigation of the possible relationship between completion rates of users of ANC services which is the dependent variable and a set of independent variables namely: provider availability, services hours, range of services, and level of service integration. Deductive techniques that are core to quantitative research were used to test the null hypothesis that there is no statistically significant relationship between provider availability, service hours, range of services, the level of service integration, and ANC completion [20].

The setting for the study were selected public and private not for profit health facilities in Iganga district in Eastern Uganda.

Using WHO’s previous standard model of ANC visits, completion of ANC attendance is defined in this study as a client having attended four or more ANC visits during her most recent pregnancy while non-completion is defined as having attended less than four ANC visits [21, 22].

### Characteristics of study participants

The study population consisted of all adult clients of reproductive age (18 – 49 years) who attended ANC services in the selected health facilities over a 12-month period prior to the study. The health workers included clinical health facility staff and/or administrative staff directly involved in management of the selected health facilities.

### Data collection procedures

The District Health Officer contacted each selected health facility in this study to communicate the research aims and logistics for data collection. The role of the District Health Officer was to provide an additional check with regard to ensuring that ethical review guidelines had been complied with prior to giving clearance for the research in Iganga district. A copy of this letter was presented to the medical officer in-charge at each facility before beginning any data collection.

The study objectives and outline were reviewed with each medical officer/health facility in-charge. Written consent was requested from the medical officer/health facility in-charge and documented as part of the study.

At all selected health facilities ANC attendance records in patient registers from the maternity departments for the twelve months prior to the study were examined. As this was a retrospective data collection process involving analysis of secondary data, the patients whose records were examined were no longer physically at the facility; instead, permission to conduct the patient record abstract data collection was sought from the medical officer in-charge at the facility. No identifiable information, such as the patient’s name, phone number, address, or otherwise was captured. The selected health facility respondents were informed that they could stop the questionnaire at any point in time without any adverse consequences.

### Variables and sources of data

The study investigated the relationship between ANC services utilization assessed as completion of four or more visits as the dependent variable and a set of independent variables namely: ANC service availability, ANC service content, and ANC service organization. Data sources included integrated ANC facility registers, summary Health Management Information System (HMIS) reports and health facility manager interviews.

### Instrumentation and measures

Data collection involved conducting structured quantitative in-person interviews with facility managers and record reviews using the a Service Level Index (SLI) tool and patient chart abstraction using a patient chart abstract tool.

#### Service level index tool

Facility assessment scores on the service level factors of interest for selected public and private not for profit health facilities were obtained using the SLI tool which was administered to the health facility in-charges. The indicators proposed for inclusion in the SLI tool are listed in Table 1. Pre-testing of the SLI tool was done as part of Study 1 of this set of studies (Level of Agreement of the SLI tool with the standard SPA tool for measurement of ANC service provision) at a selected health facility in the study district.

**Table 1:** Key parameters of ANC service provision in the service level index tool

**ANC service availability**	**Availability of trained health provider**
	Availability of medicines and medical supplies
	Availability of medical equipment
**ANC service content**	Range of recommended services provided
**ANC service organization**	Timing of services Integration with other services

#### Patient chart abstract tool

The patient abstract tool included information on the client’s age and total number of ANC visits completed. No identifiable information, such as the client’s name, phone number, address, or otherwise was captured as part of this module. To maintain client anonymity, each client enrolled in ANC over the 12-month period preceding the study was assigned a unique code/number as part of the data collection process.

At all selected health facilities, handwritten patient registers from the integrated ANC register were examined. The data in the ANC registers included data on ANC users who accessed the static ANC clinic as well as ANC outreach data tallied into the register by the health workers. Electronic health records are rarely available at health facilities in this setting except perhaps for the largest regional referral hospitals. ANC cards or Mother-Baby passports are given directly to the clients to take home and were therefore not available for review. As this was a retrospective data collection process, the patients whose records were examined were no longer physically at the facility, instead, permission to conduct the patient record abstract data collection was sought from each medical officer in-charge of a study facility.

A district that meets nationally approved criteria for classification as high burden for poverty and maternal mortality was purposively selected. Stratified random sampling was used to sample health facilities using a complete list of health facilities in the selected district as the sampling frame compiled in consultation with the District Health Management Team. Stratification ensured increased precision since the stratum are internally homogenous, but each stratum differs from the other [23].

Determination of the sample size for health records in this study was based on a standard formula for calculating an appropriate sample size for estimating magnitude/level of the dependent variable (completion of four or more ANC visits).

Based on the power analysis the study needed a minimum of three hundred seventy-four participants. Systematic random sampling was used to select these client records from the facility registers. This approach is accurate and ensured that every record had the same probability of inclusion [10].

### Statistical analysis

Logistic regression was conducted to determine the predictive relationship between ANC service availability, ANC service content, and ANC service organization and completion of ANC attendance [24]. The independent variables were ANC service availability, service content. and service organization. The dependent variable was dichotomized to create a variable with two levels: less than four visits completed, and four or more visits completed. Each of the independent variables were categorized as ordinal variables using the actual values from the SLI scores of health facilities. The regression model contained six variables (range of recommended services provided, timing of services, integration with other services, availability of medical equipment, availability of medicines & medical supplies and availability of trained health provider). Details of categorization of the variables are provided in Table 2.

**Table 2:** Categorization of Independent Variables

ANC Service Domain	Key Variables	Categorization
**ANC Service Organization**	Timing of services	Good – score equal to or more than 17
		Poor – score 10 or below (*Ref*)
	Integration with other services	Good – scores less or equal to 6 Poor—score above 6 (*Ref*)
**ANC Service Content**	Range of recommended services provided	Good – scores less or equal to 23
		Fair scores 24—25
		Poor – scores 26 or more (*Ref*)
**ANC Service Availability**	Availability of medicines and medical supplies	High- less or equal to 106
		Low—more than 106 (*Ref*)
	Availability of medical equipment	High – less than 14,
		Medium (14 – 17)
		Low—more than 17 (*Ref*)
	Availability of trained Health Provider	High—80 + % health providers trained
		Medium—50–79% health providers trained
		Low—below 50% health providers trained (*Ref*)

The relationship between the ANC completion rates of clients sampled from records at the health facilities and facility scores on service level factors of interest were analyzed. Listwise deletion was used to handle missing data. Using this approach, any cases with missing data were excluded and the remaining data were analyzed [25]. Sensitivity analysis was conducted to determine the robustness of the results to the deviations from the missing at random assumption [26]. The multiple R-statistic was used to determine the strength of the relationship between the independent variables and the dependent variable [27].

## Results

All eight study sites were public sector facilities under direct management of the government of Uganda. Six study sites offered ANC services 5 days a week, while one site offered these services 6 days a week and one site offered the services only two days a week. Seven study sites were open 24 h for routine service provision, but only one site provided ANC services on a 24-h basis. Table 3 summarizes the general characteristics of the eight study sites.

**Table 3:** Study site characteristics

Characteristic	**Total (*N* = 8)**	**HC III**	**HC IV**	**Hospital**
	8	6	1	1
Days per week the facility offers ANC services				
2 days	1	1	0	0
5 days	6	4	1	1
6 days	1	1	0	0
Hours per day the facility is open for routine services				
5 to 8 h	1	1	0	0
24 h	7	5	1	1
Hours per day the facility is open for ANC services				
5 to 8 h	4	3	0	1
9 to 16 h	3	2	1	0
24 h	1	1	0	0
Hours per day someone on call available to provide services as needed at this facility. (including the time that a facility is open for routine services)				
5 to 8 h	1	1	0	0
24 h	7	5	1	1

Under the service availability domain, only four percent of ANC completers utilized a facility with a high medical equipment availability score compared to 80% completers utilizing a facility with a low medical equipment score (*p* = 0.006), 28% of ANC completers utilized a health facility that was assigned a high availability score for medicines and medical supplies compared to 72% of ANC completers utilizing facilities with low scores for availability of medicines and medical supplies at the time the study was conducted (*p* = 0.01). For the service content domain, the majority of ANC completers (56%) utilized facilities whose range of ANC service provision was fair, but majority of non-completers utilized facilities whose range of service provision was good (*p*-value = 0.002). For the sub-domain of ANC service organization, 64% and 92% of ANC completers and non-completers respectively utilized health facilities whose timing of service provision was good (*p* < 0.001) while 24% and 54% of ANC completers and non-completers respectively utilized facilities that were practicing good integration of ANC with other services (*p* = 0.004). Table 4 summarizes the cross-tabulation findings taking ANC visit completion among ANC service users in the eight study sites between July 2019 and June 2020.

**Table 4:** ANC visit completion against key parameters of ANC service provision in service level index tool

	ANC visit completion	
	Pearson correlation	Sig. (2-tailed)
Availability of medical equipment	–.150^a^	.003
Availability of medicines and medical supplies	–.161^a^	.002
Availability of trained staff	–.163^a^	.001
Range of recommended services provided	.130^b^	.011
Timing of services	–.240^a^	.000
Integration of services	–.150^a^	.004

^a^Correlation is significant at the 0.01 level (2-tailed)

^b^Correlation is significant at the 0.05 level (2-tailed)

The full model containing all predictors was statistically significant, χ2 (6) = 26.118, *p* ˂0.05, indicating that the model could distinguish between women who completed four or more ANC visits and those who did not complete four or more ANC visits. The χ2 in the Hosmer- Lemeshow goodness of fit test was 0.732 with a significance level of 0.994 indicating a good model fit. The model as a whole explained 17.3% (Nagelkerke R squared) of the variance in ANC completion and correctly classified 93.4% of respondents. Only two of the independent variables made a unique statistically significant contribution to the model and that was availability of medical supplies and timing of ANC services. As displayed in Table 5, the results indicate that the odds of completing four or more ANC visits decrease by 0.02 for every 1 unit decrease in availability of medical supplies scores and increase by 4.6 for every 1 unit increase in ANC timing score when availability of medical equipment, trained staff, service integration and range of ANC services are controlled for (exp (B) = 0.02, *p* ˂ 0.05 and exp (B) = 4.6, *p* ˂ 0.05 respectively).

**Table 5:** Wald statistic and odds ratio

		B	S.E	Wald	df	Sig	Exp(B)	95% C.I. for EXP(B)	
								Lower	Upper
Step 1^a^	Availability of medical equipment	1.696	1.257	1.820	1	.177	5.453	.464	64.105
	Availability of medicines and medical supplies	-3.822	1.822	4.401	1	.036	.022	.001	.778
	Availability of trained health provider	-.310	.522	.353	1	.553	.734	.264	2.039
	Range of recommended services	-.547	.453	1.455	1	.228	.579	.238	1.407
	Timing of services	1.528	.595	6.600	1	.010	4.607	1.436	14.774
	Integration with other services	-1.520	1.302	1.363	1	.243	.219	.017	2.805
	Constant	8.096	2.855	8.044	1	.005	3282.854		

^a^Variable(s) entered on step 1: Availability of Medical Equipment, Availability of Medicines and Medical Supplies, Availability of trained health provider, Range of recommended services, Timing of services, Integration with other services

The prediction model contained two of the six predictors and was reached in five steps. The model was statistically significant, χ2 (2) = 22.752, *p* < 0.001, and accounted for approximately 15.2% (Nagelkerke R squared) of the variance of ANC attendance completion while correctly classifying 93.4% of study participants. As displayed in Table 6, completion of ANC attendance was primarily predicted by better timing of ANC services, and to a lesser extent by higher levels of availability of medicines and medical supplies. Timing of ANC services was found to be the most important factor for completion of ANC attendance (*p* value = 0.001). Facilities having timing of ANC service scores higher than 17 were at increased odds (4.5) of having their clients complete ANC attendance compared to those whose timing scores were less than 17. Health facilities having medicines and medical supplies scores higher than 106 which represented low availability of medicines and medical supplies in the SLI tool were at decreased odds (0.1) of having their clients complete ANC attendance compared to those whose scores were less than 106.

**Table 6:** Results of stepwise regression analysis

		B	S.E	Wald	df	Sig	Exp(B)	95% C.I. for EXP(B)	
								Lower	Upper
Step 1^a^	Availability of medical equipment	1.696	1.257	1.820	1	.177	5.453	.464	64.105
	Availability of medicines and medical supplies	-3.822	1.822	4.401	1	.036	.022	.001	.778
	Availability of trained health provider	-.310	.522	.353	1	.553	.734	.264	2.039
	Range of recommended services	-.547	.453	1.455	1	.228	.579	.238	1.407
	Timing of services	1.528	.595	6.600	1	.010	4.607	1.436	14.774
	Integration of services	-1.520	1.302	1.363	1	.243	.219	.017	2.805
	Constant	8.096	2.855	8.044	1	.005	3282.854		
Step 2^a^	Availability of medical equipment	1.386	1.144	1.468	1	.226	4.000	.425	37.659
	Availability of medicines and medical supplies	-3.677	1.808	4.134	1	.042	.025	.001	.876
	Range of recommended services	-.382	.338	1.274	1	.259	.683	.352	1.325
	Timing of services	1.363	.512	7.072	1	.008	3.906	1.431	10.664
	Integration of services	-1.065	1.069	.992	1	.319	.345	.042	2.804
	Constant	7.187	2.439	8.685	1	.003	1321.951		
Step 3^a^	Availability of medical equipment	1.386	1.144	1.468	1	.226	4.000	.425	37.659
	Availability of medicines and medical supplies	-4.058	1.776	5.220	1	.022	.017	.001	.562
	Timing of services	1.504	.506	8.852	1	.003	4.500	1.671	12.120
	Integration of services	-.924	1.066	.750	1	.386	.397	.049	3.209
	Constant	6.904	2.433	8.053	1	.005	996.148		
Step 4^a^	Availability of medical equipment	.592	.613	.931	1	.335	1.807	.543	6.014
	Availability of medicines and medical supplies	-3.264	1.491	4.794	1	.029	.038	.002	.710
	Timing of services	1.633	.495	10.891	1	.001	5.121	1.941	13.511
	Constant	5.851	2.135	7.512	1	.006	347.551		
Step 5^a^	Availability of medicines and medical supplies	-2.207	1.039	4.511	1	.034	.110	.014	.843
	Timing of services	1.507	.469	10.329	1	.001	4.511	1.800	11.306
	Constant	5.513	2.114	6.803	1	.009	247.812		

^a^Variable(s) entered on step 1: Availability of Medical Equipment, Availability of Medicines and Medical Supplies, Availability of trained health provider, Range of recommended services, Timing of services, Integration with other services

## Discussion

The study aimed to assess service-level factors possibly affecting completion of ANC attendance defined by completion of four or more visits among women of reproductive age in Iganga district in Uganda. The study investigated whether service level factors independently predict completion of ANC attendance among pregnant women in Uganda. In relation to the research hypothesis, the study demonstrated that there is a statistically significant relationship between ANC service availability, ANC service organization and completion of four or more ANC visits.

A statistically significant predictive relationship was found between timing of ANC services under the service organization domain, and completion of ANC attendance. Women were more likely to complete at least four recommended visits if they received ANC services at a health facility that reported more working hours during the day dedicated to ANC service provision and providing ANC services with higher frequency per week. These results are supported by previous findings that show that provision of ANC services on restricted days of the week presented a barrier to optimal completion of services within the ANC package such as intermittent preventive treatment in pregnancy (IPTp) [28] and the absence of scheduling, increased uncertainty among clients which in turn affected ANC attendance [29]. Timing of provision of ANC services and improved awareness among clients about what specific services in the package to expect on different days of the week has been shown to influence consistent ANC attendance [16, 30]. This finding indicates that timing of ANC services should receive increased consideration through tailored approaches which could potentially include establishment of scheduling and appointment systems for ANC visits all of which contribute to improved planning, time management and certainty from the perspective of ANC service users.

There was also a statistically significant relationship between availability of medicines and medical supplies and completion of ANC attendance. The availability of medicines and medical supplies has emerged in previous studies as a factor that influences ANC attendance. However, Ediau et al. [31], demonstrated that while improved availability of basic medical supplies for ANC service provision led to significant improvements in first ANC visit attendance, by itself this intervention was insufficient to enhance completion of at least four ANC visits. This finding under-scores the need for an increased focus on ensuring availability of medicines and medical supplies as an approach for motivating ANC service users to complete the recommended number of visits.

There was no statistically significant predictive relationship between availability of trained health providers, availability of medical equipment, range of recommended services provided, and integration of ANC services with other services, and completion of ANC attendance. Availability of trained health providers may not influence completion of ANC attendance since it has been demonstrated that provider training by itself is insufficient to improve quality of care and positive ANC experiences for clients [32]. It is plausible that the lack of a predictive relationship between availability of medical equipment, availability of trained health providers, range of recommended services and completion to ANC attendance is explained by client levels of awareness about the recommended ANC package, purpose of medical equipment for ANC and their ability to correctly gauge competence levels of health providers. Afulani et al. [33], found that 30% of ANC service users in Migori county, Kenya did not understand the purposes of tests and medicines received during ANC attendance, but this proportion decreased with increased education levels [7]. This is similar to earlier findings by Pell et al. [34], that demonstrated through qualitative interviews that while women commonly recalled receiving medicines during ANC visits, specific mentions of receipt or non- receipt of HIV and syphilis testing, hemoglobin analysis or blood pressure measurement were rarely mentioned. The authors concluded that understanding of screening / diagnostic procedures and possibly the related importance of medical equipment for these procedures was low. Furthermore, studies have shown that users of ANC services are more likely to be motivated by the perceived quality of interaction with the health provider than by the service provider’s level training and competence [32]. ANC is a platform that can facilitate the provision of multiple health interventions therefore integration with other services is considered a best practice for effective ANC service provision [35]. Some studies have found that integration of ANC services with other services as a component of service organization results in improved user satisfaction but there is limited evidence of a link between integration with other services and completion of recommended ANC visits [36, 37].

### Limitations of the study

Although this study generated new insights into the relationship between service level factors and completion of ANC attendance, there are some limitations. The low ANC completion rates in the study population translated into a very small sample size of ANC completers which may have affected the external validity of the study. Data from patient records was reviewed for the period July 2019 to June 2020. Between March 2020 and June 2020, the government of Uganda implemented strict lockdown measures that impacted utilization of routine health services across the country due to restrictions on the use of public transportation. This factor was not controlled for during analysis but may have affected the continued utilization of ANC services among women whose records were reviewed. This limitation as well as the short data period of one year and the focus on only one district may affect generalizability of the results.

## Conclusions

This study has demonstrated that service level factors have a predictive value for completion of ANC attendance. The findings can be used to improve availability, content, and organization of ANC services with the aim of enhancing positive experiences for clients and motivating them to complete the recommended number of visits. Timing of ANC service provision, as well as the availability of medicines and medical supplies for ANC service provision, should be prioritized by managers at different levels of the health system. Further research is needed to improve measurement of service level factors and how these interact with ANC service completion incorporating perceptions of service users themselves.

### Supplementary Information

**Additional file 1. **

**Additional file 2. **

## Data Availability

The datasets used and/or analysed during the current study available from the corresponding author on reasonable request.

## Abbreviations

ANC: Antenatal Care
HMIS: Health Management Information System
SLI: Service Level Index
SPA: Service Provision Assessment
WHO: World Health Organisation
